# Energy, exergy, exergo-economic, enviro-economic, exergo-environmental, exergo-enviro-economic, sustainability and sensitivity (6E,2S) analysis on single slope solar still—An experimental study

**DOI:** 10.1371/journal.pone.0290250

**Published:** 2023-08-24

**Authors:** Muhammad Alam Zaib Khan, Abdul Wahab, Kamran Khan, Naveed Ahmad, Muhammad Ali Kamran

**Affiliations:** 1 Mechanical Engineering Department, University of Engineering and Technology, Peshawar, Peshawar, Pakistan; 2 Thermal System Engineering Department, University of Engineering and Technology, Peshawar, Peshawar, Pakistan; King Fahd University of Petroleum and Minerals, SAUDI ARABIA

## Abstract

Tackling water scarcity is a significant challenge due to the rapid increase in the global population, which is raising concern for the supply of fresh water. high demand of fresh water leading to a failure in meeting the demand for fresh water. This study aims to investigate the feasibility of an efficient single-slope solar still with an aluminum-finned plate absorber and internal and external reflectors to address water scarcity. Energy, exergy, economic and environmental analyses (6E) were undertaken to deeply analyze its impact on the environment. The maximum energy and exergy efficiency achieved was 60.19% and 21.57%, respectively, at a 2cm depth. The use of both external and internal reflectors assisted in the highest productivity of 7.02 liters. The cost of 0.033$/liter was obtained for a lifetime of 10 years for the optimal system. The payback time in terms of energy and exergy for the optimal system is 0.88 and 2.23 years, respectively. Furthermore, sustainability and sensitivity (2S) analysis were also performed to assess the system’s current and future feasibility. The total price for carbon dioxide mitigation during the solar still lifetime was $346.7, which represents the cost saving achieved with the installation of the optimal system.

## 1. Introduction

Despite the earth surface covers 71% of water of the total area, the challenges to meet the freshwater demand are still stiff of humans, animals and plants. The earth surface contains 97.5% saline water of the total water and the remaining 2.5% is the fresh water which is in the form of ground water and icebergs [1]. The accessible water contributes only by 0.008% of the total fresh water. That is why due to less availability of fresh water large population around the globe suffers water scarcity [2]. The demand of fresh water increases day by day due to rapid increase in population and industrialization. The natural available fresh water resources fail to meet the excessive fresh water demand. The water scarcity occurs when water supply falls below the recommended standard which is 1000 m^3^ /person/year [3]. According to the United Nations Organization that around the world approximately 1.8 billion people would be the under utmost scarcity of water by 2025 [4]. The significance of fresh water can be examined by narrative of UNO Secretary General on world water day 2002 that almost 1100 million people around the world have insufficient approach to potable water, almost 2500 million people lack in proper sanitation of water and almost more than 5 million people die due to waterborne diseases each year [5]. Water management is crucial for economic development. Abba et al. [6] studied Yumna water quality considering agricultural, industrialization and urbanization development at Yumna basin. It was concluded that due to anthropogenic activities in this area, Yumna water is only suitable for industrial cooling and agriculture. Measures were suggested to maintain the healthy ecosystem which includes the restriction of drainage and disposal of waste into the river and industrial treatment of effluents for abatement of pollution in future. In another study [7], socioeconomic development models were undertaken with AI based methods. Thus, a new approach was developed which involves the use of direct modelling approach to understand the behavior of various independent variables for ground water salinization. In this model, input variables can also be combined with influential factors for deep understanding of ground water salinization. Ground water salinization mapping was also performed in another study by Abba et al. [8] where meta heuristic modelling algorithms were used to study the alarming socio-economic menace in various places like Saudi Arabia.

Desalination is the process of elimination of salts and minerals from salty water to make it potable. There are many conventional methods used for the desalination of water worldwide, the most common includes electro dialysis, reverse osmosis, multi effect distillation, multi stag flash and vapor compression [9]. It is of great significance to be aware of the energy used by the desalination processes to acknowledge why renewable and sustainable desalination technologies should be adopted for desalination of water. Although conventional desalination systems are efficient but have environmental impacts due to emission of toxic and harmful gases. It leads to increase the environment temperature which intends to global warming. The effect of greenhouse by conventional desalination processes can be estimated from the quantity of fossil fuels used for desalination of water. For the production of 1x10^6^ liters of potable water by desalination processes almost 5000 Kilograms fossil fuels are used which results in the production of 5000 m^3^ of greenhouse gases [10]. Conventional methods use fossil fuels directly or indirectly for power supply which causes green house effect. Many researchers are focused to mitigate the dependency of desalination technologies on fossil fuels by integrating desalination technologies with renewable energy [11]. Renewable energy desalination is most applicable to rural, remote and those areas where there is no mean of energy other than renewable energies [12]. Solar, geothermal and wave energies are the most commonly used renewable energies used for desalination process, but among these solar energy contribute 57% to desalination of water which is highest among all [13].

Solar still is the most viable and simple technique to make potable water from saline water, but it has lower productivity [14]. Many factors influencing the performance of solar still are categorized in climatic, design and operational parameters. The climatic parameters include solar radiation, cloud and dust cover, ambient temperature, relative humidity and wind velocity. The evaporation area, cover glass inclination angle, water depth, tracking and reflectors, thermal storage materials and additives and insulation are the deign parameters effecting performance of solar still. Similarly the operational parameters include water feeding, position of the solar still and maintenance of the solar still [15–19].

The aluminium and galvanized iron have used by many researchers as an absorber plate. Anburaj et al. [20] experimentally studied the performance of solar still with aluminium having thickness of 1 mm and black coated as an absorber plate. The productivity of 3.76 liter/m^2^ has achieved in sunny days. Sengar et al. [21] used Galvanized Iron as an absorber plate with corrugated surface area of 1 m^2^ coated with black paint in order to have a maximum absorptivity of solar radiation. The efficiency of 56.3% in summer and 47.14% in winter has been recorded.

The water depths in the solar still has significant effect on the performance of the solar still and get keen attention for researchers. The productivity of solar still decreases by increasing water depth. Many researchers [22–24] examined the effect of water depths on the productivity of solar still and concluded that by increasing water depth the productivity of solar still decreases during the day time and vice versa. A. Ahsan et al. [25] designed and fabricated low cost solar still for offshore rural areas for the production of potable water from saline water. The productivity was investigated at depth of 1.5, 2.5 and 5 cm and concluded that productivity and water depth have inverse relation. In the study of Khalifa et al. [26] the performance of solar still were examined with variation in the depth of water that is 1, 4, 6, 8, and 10 cm. It was deduced that productivity decreased by 48% by increasing the depth of water. But increase in the depth of water during the night time increased the productivity and hence increased the daily output. By many researchers the recommended water depth ranges from 2 to 6 cm [27].

The performance of solar still with reflectors has reviewed by Omara et al. [28]. It has concluded that reflectors would be beneficial especially in those areas where the solar radiation is lower. Hayek et al. [29] comparatively studied the performance of simple solar still and double slope solar still in the month of August. The insides wall of the simple solar still were covered with mirrors. The productivity of simple solar still has risen by 20% as compared to double slope solar still. Karime et al. [30] carried theoretical and experimental study on the performance of solar still with reflectors in summer and winter seasons. It has been deduced that productivity increased by 18% upon installation of internal reflectors on front and side walls. They further added that performance of solar still with internal reflector on back wall has increased by 22% annually. The productivity increased upon installation of internal reflectors by 65%, 22%, and 34% in the season of winter, summer and whole year respectively as compared to solar still without internal reflectors. Tanaka et al. [31] investigated the performance of conventional solar still with internal and external reflectors in winter season. The results revealed that productivity could be increased up to 100% using internal and external reflectors than that of simple solar still. Khalifa et al. [32] experimentally studied the effect of internal and external reflectors with an inclination angle of 0°, 10°, 20° and 30° on the productivity of solar still. The study revealed that yield increased by 19.7% using internal reflectors. The distillate increased by 34.5%, 34.4%, 34.8%, and 24.7% using external reflectors at an inclination angle of 0°, 10°, 20°, and 30° respectively.

A lot of research has been conducted on the exergy analysis of solar stills in the past. Yousef et al. [33] conducted exergy analysis on solar still using different absorbing materials with hollow cylindrical pin fins. Maximum exergy efficiency of 23% was achieved. It involves energy, exergy, economic and environmental analysis. However, this study lacks sustainability analysis, exergo-environmental and exergo-enviro-economic analysis. In another research, Yousef et al. [34] conducted energy and exergy analysis on single slope solar still which lacks environmental and sustainability analysis. It lacks the methodology to access the carbon pricing and carbon mitigation. Dumka et al. [35] studied single slope conventional solar still to analyze the energy, exergy and environmental analysis. This study used ultrasonic fogger and cotton cloth to enhance the efficiency of the system. However, this study lacks sustainability analysis to analyze the multiple factors like improvement potential and sustainability index to better analyze the system performance. In addition to this, no exergo-environmental and carbon mitigation study was done. Parsa et al. [36] conducted a very detailed study on solar still water desalination. However, this study still lacks more insights for accurate carbon pricing which involves exergo-enviro-economic analysis as stated by Caliskan [37]. Furthermore, no sensitivity analysis has been discussed to predict the future scenarios.

In this research, experimental work has been carried on single basin fin plate absorber solar still integrated with internal and external reflectors under Pakistan climate. Previous studies has mostly focused on energy, exergy, economic and environmental analysis on solar still. No studies have been conducted to describe the sustainability analysis, sensitivity analysis and exergo-enviro-economic (EXENEC) analysis which determines a major research gap. This study along with energy, exergy, economic and environmental analysis also undertook the sustainability analysis, sensitivity analysis and exergo-enviro-economic (EXENEC) analysis to bridge the knowledge gap which states a novelty of this research. Sustainability analysis is extended to determine the system feasibility in terms of improvement potential and sustainability index to determine the performance of the system and the extent to which system performance can be increased to get full use of available energy. It also determines payback period which has been a part of previous studies. Furthermore, sensitivity analysis is performed to intercept the future predictions which to the best of author’s knowledge is not analyzed before for solar desalination process. It determines the feasibility of the system considering the change in parameters which can directly affect the system cost and performance. Moreover, exergo-enviro-economic (EXENEC) is undertaken for promising methodology to access the carbon pricing effectively with the help of life cycle and enhanced thermodynamics [37]. This study also involves water depth and external reflector as a variable to analyze its impact on the overall system performance. Results has been visualized on graphs for 6E,2S analysis on single slope solar still desalination.

## 2. Methodology

### 2.1 Experimental setup

**Fig 1:**
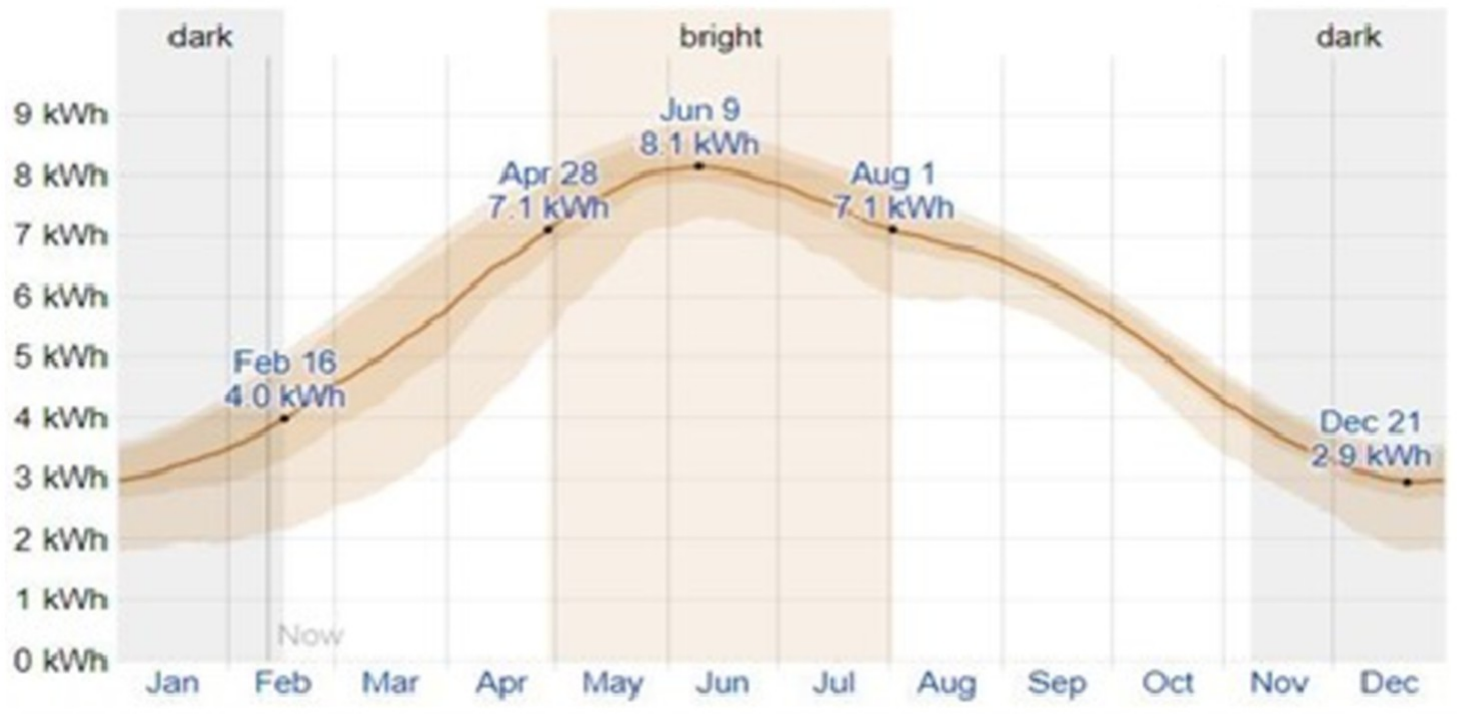
Solar radiations though out the year in Peshawar [38].

The experimental work was carried at U.S-Pak Center for Advanced Studies in Energy, University of Engineering and Technology (UET) Peshawar, Pakistan. Solar radiation data was acquired from weather station installed at UET Peshawar, as the productivity of solar still is highly dependent on the solar radiation. Peshawar has a variable solar intensity from 2.9 kWh/m^2^ to 8.1 kWh/m^2^ with a mean value of 4.5 kWh/m^2^/d, as shown in Fig 1.

**Fig 2:**
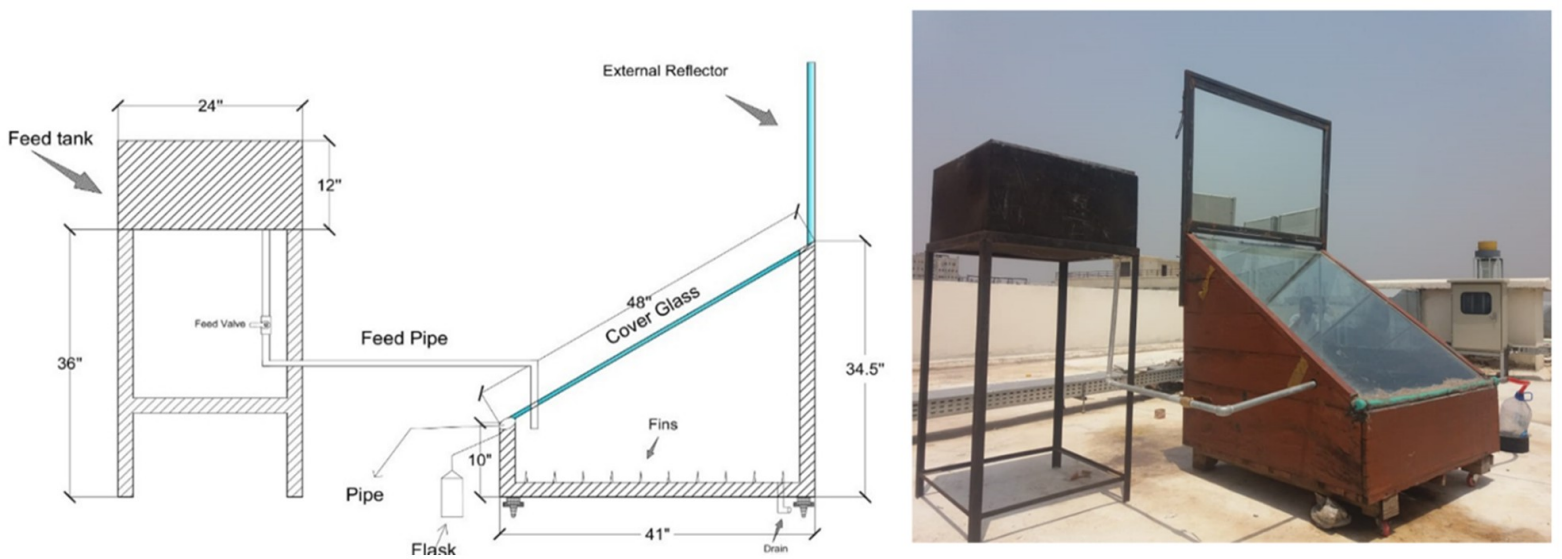
Schematic diagram of solar still and photographic view of the setup.

The schematic and experimental setup is shown in Fig 2. Aluminium has been used as an absorber plate with basin area of 1 m^2^ with a height of 20.32 cm. The 64 numbers of fins made of aluminum with height of 6.35 cm and diameter of 2 mm have placed with a pitch of 10.16 cm in the basin. The basin is coated with black coating to have maximum absorptivity of solar radiation. Thermocole with thickness of 2.54 cm has been used as an insulating material to prevent heat losses. A wooden box with dimensions of 1.08 m^2^ base area, 0.87 m back side wall height and 0.254 m front wall height has used in order to support an absorber plate of area 1 m^2^. The base is covered with transparent glass of 8 mm in thickness at an inclination angle of 30° to ensure maximum radiations to the still basin and smooth flow of condensate. The two side walls and back wall inside the still have covered with mirror of 5 mm thickness to be used as an internal reflector (I.R). A plane mirror of 0.60 m^2^ and 5 mm in thickness has been used as an external reflector (E.R). A movable steel frame has been used for an external reflector to focus the solar radiation into the basin. A feed tank made of galvanized iron with area of 0.37 m^2^ and height of 30.48 cm is used for the feeding of saline water. Tank was placed on the frame of height 0.91 m in order to have a smooth flow of the saline water to the still. The solar still specifications are given in Table 1. The ambient temperature, basin water temperature and still inside temperature have been measured by means of K-type temperatures sensors linked with data logger after interval of 1 hour from 08:00 hours to 17:00 hours. Similarly, the productivity has been measured in calibrated flask after 1-hour interval. Saline water of 35 g/l has been used in all the experiments.

**Table 1:** Solar still components specifications.

Component	Specification
Wooden box	Area = 1.08 m^2^, Back wall height = 0.87 m, Front wall height = 0.254 m
Absorber plate (Al)	Area = 1 m^2^, wall heights = 20.32 cm,
Fins	Number = 64, Pitch = 10.16 cm
Insulation	Material = Thermocole, Thickness = 2.54 cm
Cover glass	Material = Transparent glass, Thickness = 8 mm, Inclination angle = 30°
Reflectors	Material = Mirror, Thickness = 5 mm, E.R area = 0.60 m^2^, 3 I.Rs
E.R frame	Type = Moveable, Height = 0.96 m, Width = 1.09 m
Feed water tank (GI)	Material = G.I Sheet Dimensions = 0.60x0.60x0.30 m
Feed water tank stand	Height = 0.91 m, Width = 0.61 m

The experiments have been carried in four sets as mentioned below:

- **Case A**: SS with internal reflectors and basin depth of 2 cm
- **Case B**: SS with internal reflectors and basin depth of 4 cm
- **Case C**: SS with both internal and external reflectors and basin depth of 2 cm
- **Case D**: SS with both internal and external reflectors and basin depth of 4 cm

### 2.2 Formulas for data interpretation

The calculations for thermodynamics, sustainability, economic and environmental analysis were done as follows:

#### 2.2.1 Thermodynamic analysis

*Energy analysis*

Thermal efficiency ƞ_*th*_ is calculated based on hourly fresh water production using Eq (1) [33].

$${ƞ}_{th}=\frac{{\dot{m}}_{w}h_{fg}}{I_{s}A_{b}\Delta t}$$ (1)

where, ${\dot{m}}_{w}$ denotes freshwater production (m^3^/h), *h*_*fg*_ is latent heat of evaporation of water (J/kg), *I*_*s*_ is the solar intensity (W/m^2^), *A*_*b*_ is the solar still area (m^2^) and Δ*t* represents the time interval in seconds while calculating the efficiency.

*Exergy analysis*

Exergy efficiency ƞ_*ex*_ shows the useful work which can be acquired from solar still using Eq (2) [34].

$${ƞ}_{ex}=\frac{{\dot{E}}_{x,out}}{{\dot{E}}_{x,in}}$$ (2)

where, ${\dot{E}}_{x,out}$ is the exergy output from solar still and ${\dot{E}}_{x,in}$ is exergy input in solar still which can be calculated as follows [37, 39]:

$${\dot{E}}_{x,out}={\dot{m}}_{w}h_{fg}\left[1-\left(\frac{T_{amb}}{T_{w}}\right)\right]$$ (3)

$${\dot{E}}_{x,in}=I_{s}A_{b}\left[1-\frac{4}{3}\left(\frac{T_{amb}}{T_{s}}\right)+\frac{1}{3}{\left(\frac{T_{amb}}{T_{s}}\right)}^{4}\right]$$ (4)

where, *T*_*amb*_ is the ambient temperature, *T*_*w*_ is the water temperature and *T*_*s*_ is the surface temperature of the solar still.

*Exergo-economic analysis*

Exergo-economic parameters for exergy and energy can be calculated as follows [33]:

$$R_{Ex}=\frac{{\dot{Ex}}_{out,\;ann}}{TAC}$$ (5)

$$R_{En}=\frac{{\dot{En}}_{out,\;ann}}{TAC}$$ (6)

Where *R*_*Ex*_ and *R*_*En*_ represents the exergy and energy exergo-economic parameters respectively. Similarly, ${\dot{Ex}}_{out,ann}$ and ${\dot{En}}_{out,ann}$ are annual exergy and energy outputs (kWh) respectively for solar still and TAC represents the total annual cost which is calculated as follows [35]:

TAC=FAC+AMC−ASV (7)

Where AMC is the annual maintenance cost which is taken as 10% of FAC as mentioned by Shoebi et al. [40]. FAC represents first annual cost of solar still which can be determined as follows [41]:

$$FAC=C_{c}*CRF$$ (8)

Where *C*_*c*_ is the capital cost of solar still and CRF denotes the capital recovery factor which can be calculated as follows [42]:

$$CRF\left(i,n\right)=\frac{i{\left(1+i\right)}^{n}}{{\left(1+i\right)}^{n}-1}$$ (9)

Where i is the interest rate which is 13% and n is the total lifetime of the solar still which is taken as 10 years in this study. Furthermore, yearly salvage value ASV is calculated as follows [35]:

ASV=S*SFF (10)

Here S is the salvage value of the solar still which is taken as 20% of the capital cost (C_c_) as mentioned by Shoeibi et al. [40] and SFF is the sinking fund factor which can be determined as [41]:

$$SFF=\frac{i}{{\left(1+i\right)}^{n}-1}$$ (11)

#### 2.2.2 Sustainability analysis

*Energy payback time (EPBT)*

Energy payback time (EPBT) is the time required by output of solar still to retrieve the energy consumed by parts of solar still during manufacturing process. EPBT value is based on energy and exergy annual output and can be calculated as follows [43]:

$$EPBT_{En}=\frac{E_{in}}{{\dot{En}}_{out,ann}}$$ (12)

$$EPBT_{Ex}=\frac{E_{in}}{{\dot{Ex}}_{out,ann}}$$ (13)

Where *E*_*in*_ is embodied energy, *EPBT*_*En*_ and *EPBT*_*Ex*_ are energy payback time for energy and exergy respectively.

*Energy Production Factor (EPF)*

Energy production factor (EPF) is the inverse of energy payback time and can be calculated as follows based on energy and exergy [36]:

$$EPF_{En}=\frac{{\dot{En}}_{out,ann}}{E_{in}}$$ (14)

$$EPF_{Ex}=\frac{{\dot{Ex}}_{out,ann}}{E_{in}}$$ (15)

*Waste exergy ratio*

Waste exergy ratio (WER) is determined as the amount of energy that is lost during the process. Exergy waste is the difference between exergy input and output in the solar still. However, this exergy waste can be reduced by reducing exergy losses in the system [33].

$$\text{WER}=\frac{Ex_{waste}}{Ex_{in}}$$ (16)

Where *Ex*_*waste*_ is the annual exergy waste in solar still.

*Environmental effect factor*

Environmental effect factor (EEF) determined the impact of exergy waste on the environment and it can be calculated as follows [44]:

$$\text{EEF}=\frac{WER}{{ƞ}_{ex}}$$ (17)

*Improvement potential*

Improvement potential (IP) determines the extent to which the potential of solar still system can be enhanced. It can be calculated as follows [37]:

$$\text{IP}={\left(1-{ƞ}_{ex}\right)}^{2}*Ex_{in}$$ (18)

*Sustainability index*

Sustainability Index (SI) indicates the performance of solar still system based on the utilizable system efficiency. It can be calculated as follows [45]:

$$\text{SI}=\frac{1}{1-{ƞ}_{ex}}$$ (19)

#### 2.2.3 Environmental impact analysis

*CO_2_ emission*

Carbon dioxide emission during solar still lifetime can be calculated as follows [40]:

$$LCDE=X_{CO_{2}}=2*E_{in}$$ (20)

$$ACDE=\frac{2*E_{in}}{n}$$ (21)

Where LCDE represents lifetime carbon dioxide emission and ACDE indicates annual carbon dioxide emissions.

*CO_2_ emission mitigation*

Net carbon dioxide mitigation during solar still life time can be calculated as follows [46]:

$$\emptyset_{CO_{2}}=\frac{2*\left(\left(E_{en}{)}_{out}*n\right)-E_{in}\right)}{1000}$$ (22)

Where $\emptyset_{CO_{2}}$ is the environmental parameter.

*Enviro-economic (ENEC) analysis*

Enviro-economic analysis represents the price of net carbon dioxide mitigation during solar still lifetime. It can be determined as follows [37]:

$$Z_{CO_{2}}=X_{CO_{2}}*\emptyset_{CO_{2}}$$ (23)

Where $Z_{CO_{2}}$ represents the enviro-economic parameter and $X_{CO_{2}}$ is the international carbon price.

*Exergo-environmental (EXEN) analysis*

Exergy-environmental analysis displays the net reduction in carbon dioxide emissions for energy consumption during water production using renewable resources instead of fossil fuels. It can be evaluated as follows [36]:

$$\emptyset_{ex,CO_{2}}=\frac{2(\left(E_{ex}{)}_{out}*n-E_{in}\right)}{1000}$$ (24)

Where $\emptyset_{ex,CO_{2}}$ is the exergo-environmental parameter in tons.

*Exergo-enviro*-*economic (EXENEC) analysis*

Exergo-enviro-economic analysis determines the price of carbon dioxide mitigation considering exergy for solar still lifetime. It can be determined as follows [37]:

$$Z_{ex,CO_{2}}=X_{CO_{2}}*\emptyset_{ex,CO_{2}}$$ (25)

Where $Z_{ex,CO_{2}}$ is the exergo-enviro-economic parameter.

## 3. Results and discussion

The performance of solar still was investigated with varying the depth of water, using internal reflectors and combination of internal and external reflectors. The experiments were carried out in four sets, i) using only internal reflectors at depth of 2 cm. ii) using both internal and external reflectors at depth of 2 cm. iii) using only internal reflectors at depth of 4 cm. iv) using both internal and external reflectors at depth of 4 cm.

### 3.1 Ambient temperature and productivity

**Fig 3:**
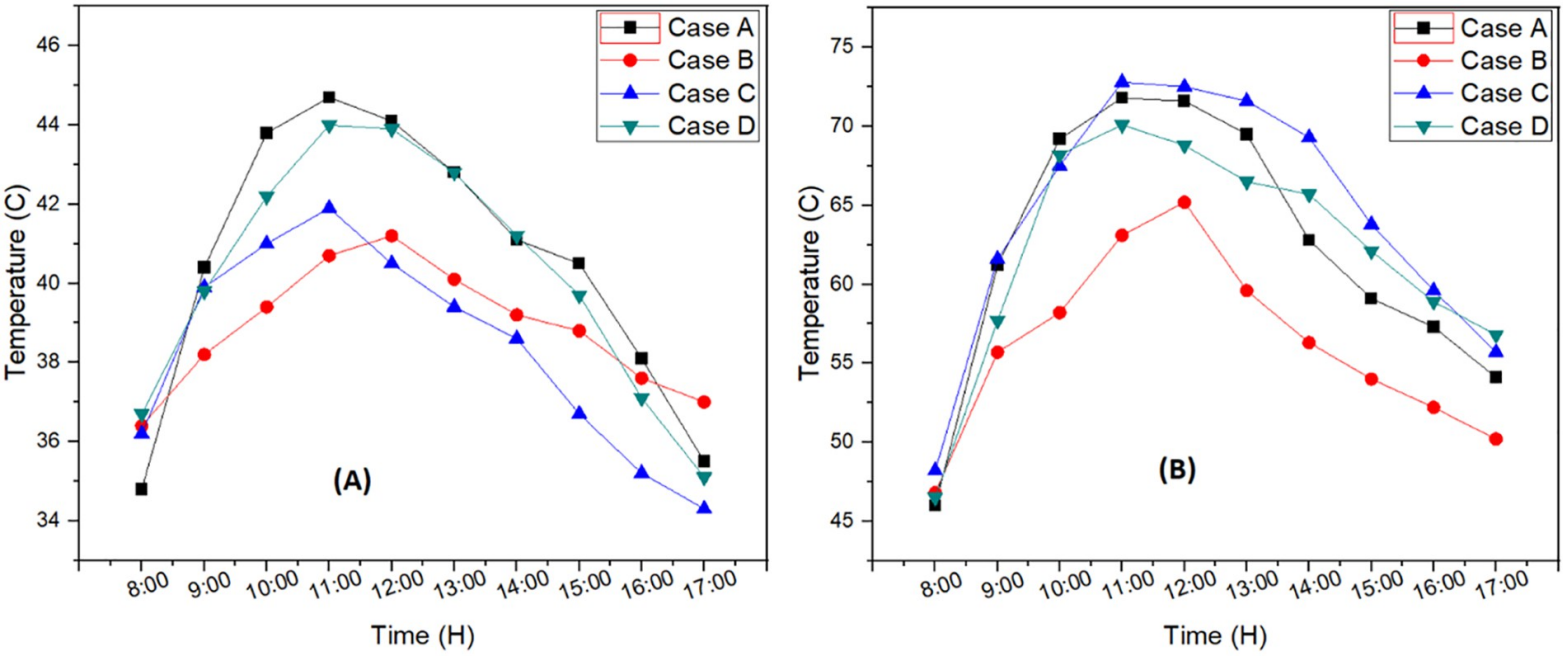
(A) Ambient temperature for each case (B) Basin water temperature for each case.

Experimentation was conducted at Peshawar, Pakistan during hot days of summer to optimize the productivity of solar still considering various parameters. Ambient temperature from 8:00 AM to 5:00 PM during experimentation is shown in Fig 3A. Experimental setup was installed at the facility of Mechanical Engineering Department, University of Engineering and Technology Peshawar, Pakistan. The maximum and minimum peak temperature observed was 44.7°C and 41.2°C.

The temperature of water inside the solar still was also recorded using temperature sensor which is shown in Fig 3B. Case C showed 7.3 °C higher temperature compared to Case B because of addition of external reflectors and decrease in basin depth. External reflector directed more solar radiations towards basin water which resulted in higher water temperatures leading to more productivity. The maximum basin temperature observed was 72.5°C, 71.8°C, 70.1°C and 65.2°C for case C, case A, case D and case B, respectively. System was optimized by varying the parameters to get maximum output efficiency.

**Fig 4:**
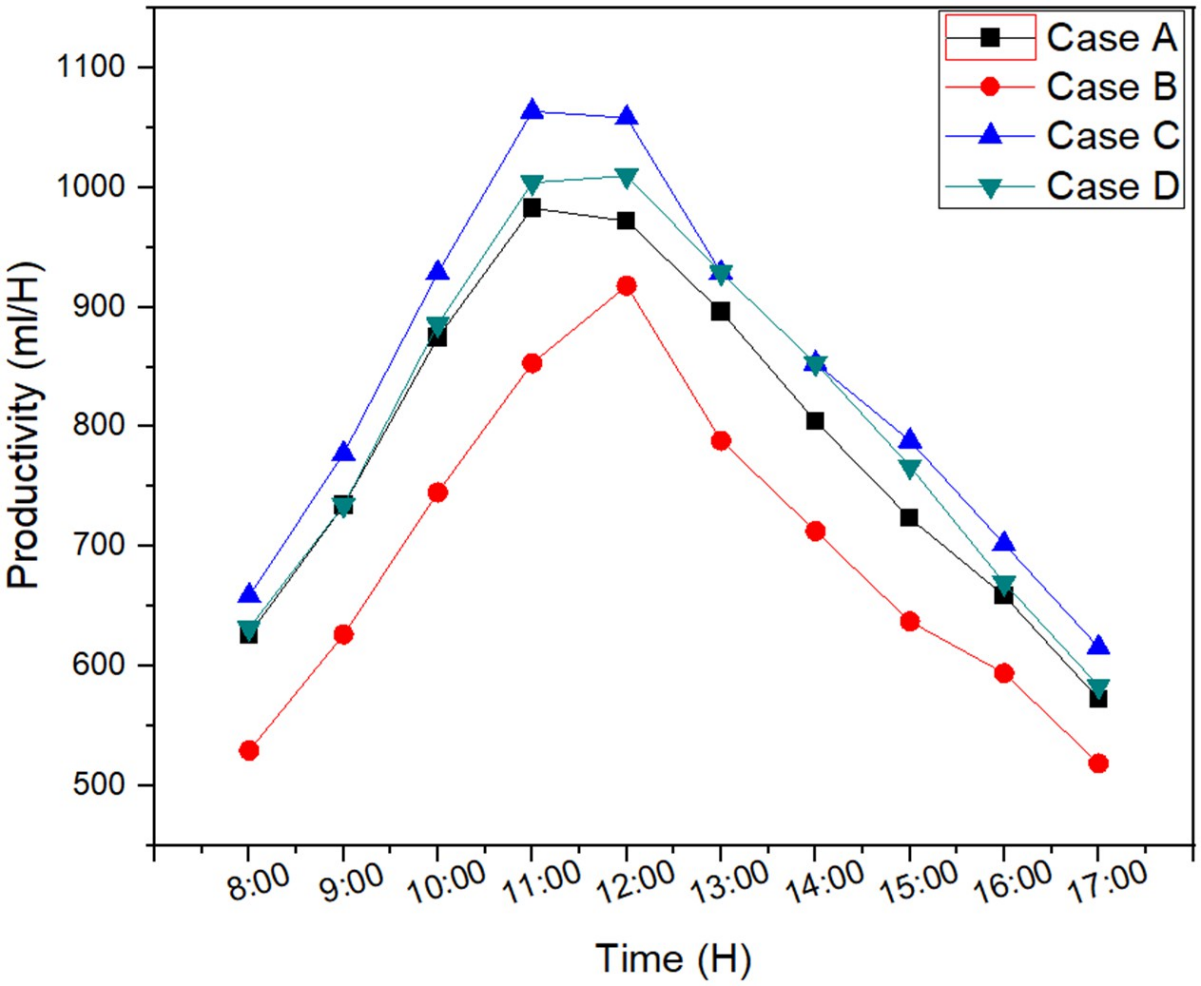
Productivity for each case; Case A: SS with internal reflector and basin depth of 2 cm; Case B: SS with internal reflector and basin depth of 4 cm; Case C: SS with both internal and external reflectors and basin depth of 2 cm; Case D: SS with both internal and external reflectors and basin depth of 4 cm.

Productivity for each case was noted hourly which is shown in Fig 4. Case C had 17.3% higher water productivity compared to case B which was due to higher solar intensity available by using case C, compared to case B and A. The use of internal and external reflectors enhanced the available energy to the solar still to optimize the productivity by raising the basin temperature. However, the result showed that water productivity was reduced by increasing the water depth as observed in case D. The water productivity declined by 3.4% by raising the water depth from 2 cm (Case C) to 4 cm (Case D). Reason for this decline was the temperature difference between the glass inside and basin water got worse with the increase in basin depth. Maximum productivity of 8.37 liter was observed for case C followed by case D with 8.06 liter, case A with 7.84 liter and case B with 6.92 liter per day. Thus, the addition of external reflector in desalination process and reduction in basin depth resulted in more productivity.

The productivity of water was directly related to solar radiation. The energy available to raise the basin temperature was increased with increment in solar radiation up till 12:00 PM. As soon as the solar radiation had a declining trend, energy of the system was reduced, which significantly affected the basing temperature, ultimately reducing the water productivity. Maximum productivity was achieved during noon 1058.4 ml/H.

### 3.2 Energy and exergy analysis

**Fig 5:**
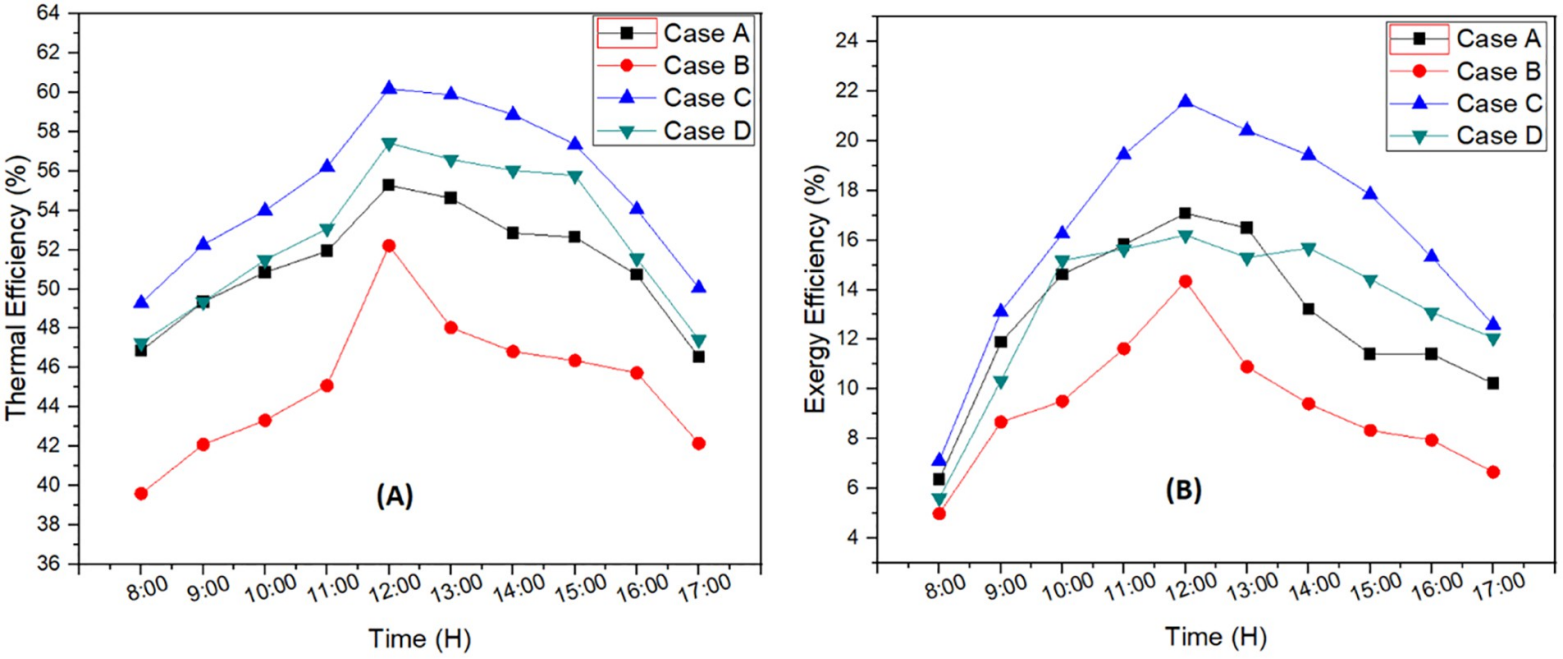
Thermal efficiency (A) and exergy efficiency (B) for each case.

Thermal efficiency for each case is shown in Fig 5A. The maximum efficiency at peak temperature was 60.19%, 57.43%, 55.28% and 52.21% for case C, case D, case A, and case B respectively. The use of external reflector at 2cm depth resulted in 8.88% rise in efficiency. Furthermore, due to decrease in depth of basin from 4 cm to 2 cm resulted in 4.8% increase in thermal efficiency. It is because the temperature difference between water and glass inside area is improved for basin with 2cm and integration of both reflectors as it helps to absorb more solar radiation. Hence, the optimal system was declared at 2cm depth of basin and use of both internal and external reflectors i.e., case C. Moreover, further analysis was conducted to indicate the losses and improvements within the system.

Exergy efficiency was calculated to know how much useful energy is available to the system for certain application such as solar still. Maximum peak exergy efficiency achieved was 21.57% for case C followed by case A (17.03%), case D (16.20%), and case B (14.33%) which can be seen Fig 5A. The exergy efficiency was increased by 33.14% by reducing the depth to 2 cm from 4 cm, while addition of external reflector resulted in 26.28% rise in efficiency. Thus, a great change in exergy efficiency was observed by changing the design parameters. Addition of reflectors resulted in great temperature change, leading to more production of clean water. Furthermore, decrease in height for basin resulted in improved exergy efficiency which can also be seen in the study conducted by Khafaji et al. [47]. Case C was declared as the optimal system in the present study. Optimal system resulted in 26.28%, 50.52% and 33.14% better performance compared to case A, case B and Case D respectively.

### 3.3 Economic analysis

The cost of the solar still should be kept lower to provide distillate water at lower cost as compared to other desalination techniques. The total fixed cost of the studied solar still integrated with internal and external reflectors is about 150$ as shown in Table 2.

**Table 2:** Cost estimation.

Material	Price($)
Wooden box	24
Aluminum absorber plate	31
Mirrors	30
G.I storage tank	10
Cover glass	29
Ball valve	6
Paint	3
Thermocole	2
G.I pipes	7
External reflector stand	3
Miscellaneous	5

According to the cost analysis formula [48],

C=F+V (26)

Here F is the fixed cost and V is the variable cost. Assuming variable cost of V = 0.3F per year and life time of 10 years for the solar still.

Then *C* = 150 + 0.3 * 150 * 10 = 600$

Assuming minimum sunny days of 300 in one year and the average productivity of solar still with internal and external reflectors 6 liters/day. Then the productivity for 10 years would be 6x10x300 = 1800 liters. The cost of 1 liter would be 600$ **/** 1800 liters = 0.033$/liter. However, cost of water for Yousef et al. [33] was 0.0416$/liter and 0.034$/liter for the optimal system with the use of hollow cylindrical pin fins and steel wool fibers in single slope solar still respectively. In the present study, only rectangular fins were used as absorbing material.

### 3.4 Exergo-economic analysis

Exergo-economic analysis of solar still provide a cost effective solution while considering exergy output and economic analysis. Uniform annual cost for optimal system calculated was 22.48 $. Considering the average output, the results were extended for whole year based on the results of experimentation in order to make the realistic results. The exergo-economic parameter for energy and exergy was calculated as 22.05 $ and 8.74 $ respectively considering the optimal system having 2cm basin depth and use of internal and external reflector. The exergo-economic parameters were enhanced for optimal system due to higher output which consequently resulted in low water production cost compared to other cases.

### 3.5 Sustainability analysis

**Fig 6:**
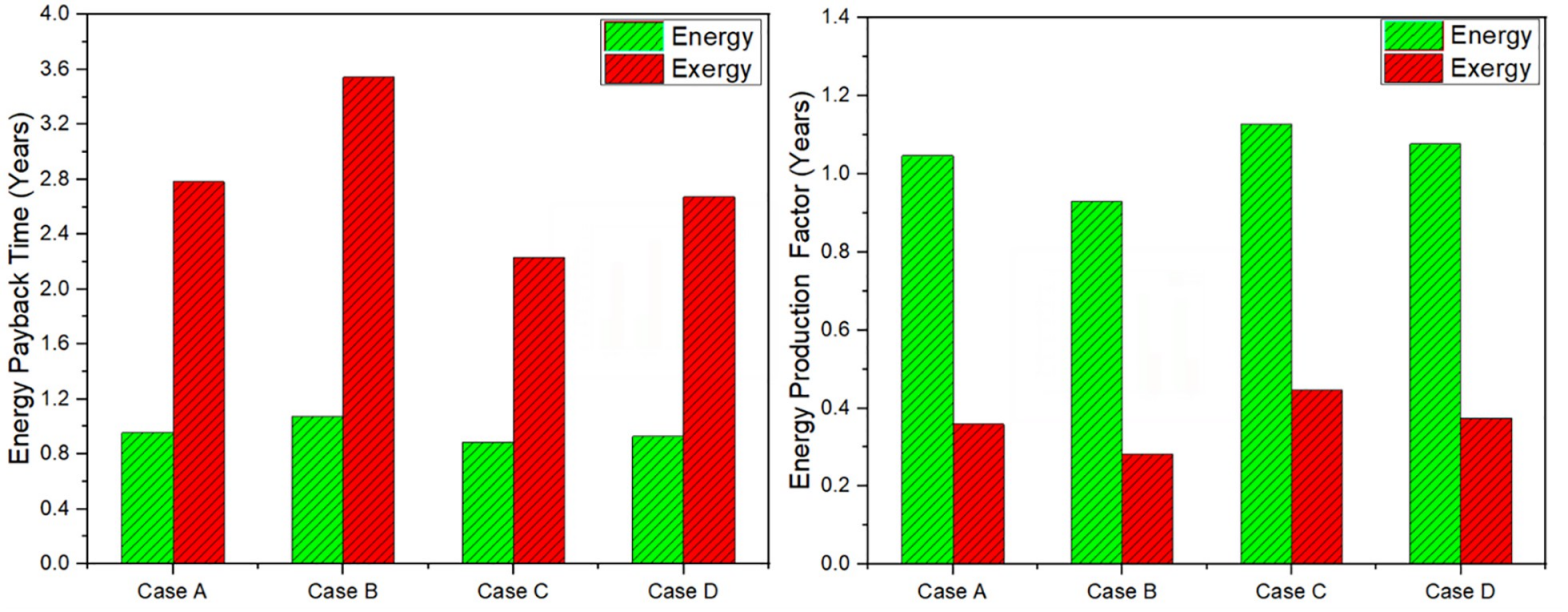
Energy payback time (left bar chart); Energy production factor (right bar chart) for each case.

Energy payback time indicates the time required to recover the solar still embodied energy. Fig 6 shows the energy payback period of each case considering energy and exergy output. Considering exergy output, payback period is quite higher compared to energy output. Case B showed maximum payback period in terms of both exergy and energy output as 3.54 and 1.07 years respectively. However, minimum payback time period was for case C as 0.88 and 2.23 years for energy and exergy output respectively. However, the energy payback period time as per Parsa et al. [36] study was 1.57 years. Energy production factor determines the overall performance of solar still. The Fig 6 also shows the measured performance of each case based on energy and exergy output. Maximum overall execution was for case C for both energy and exergy outputs and minimum for case B.

**Fig 7:**
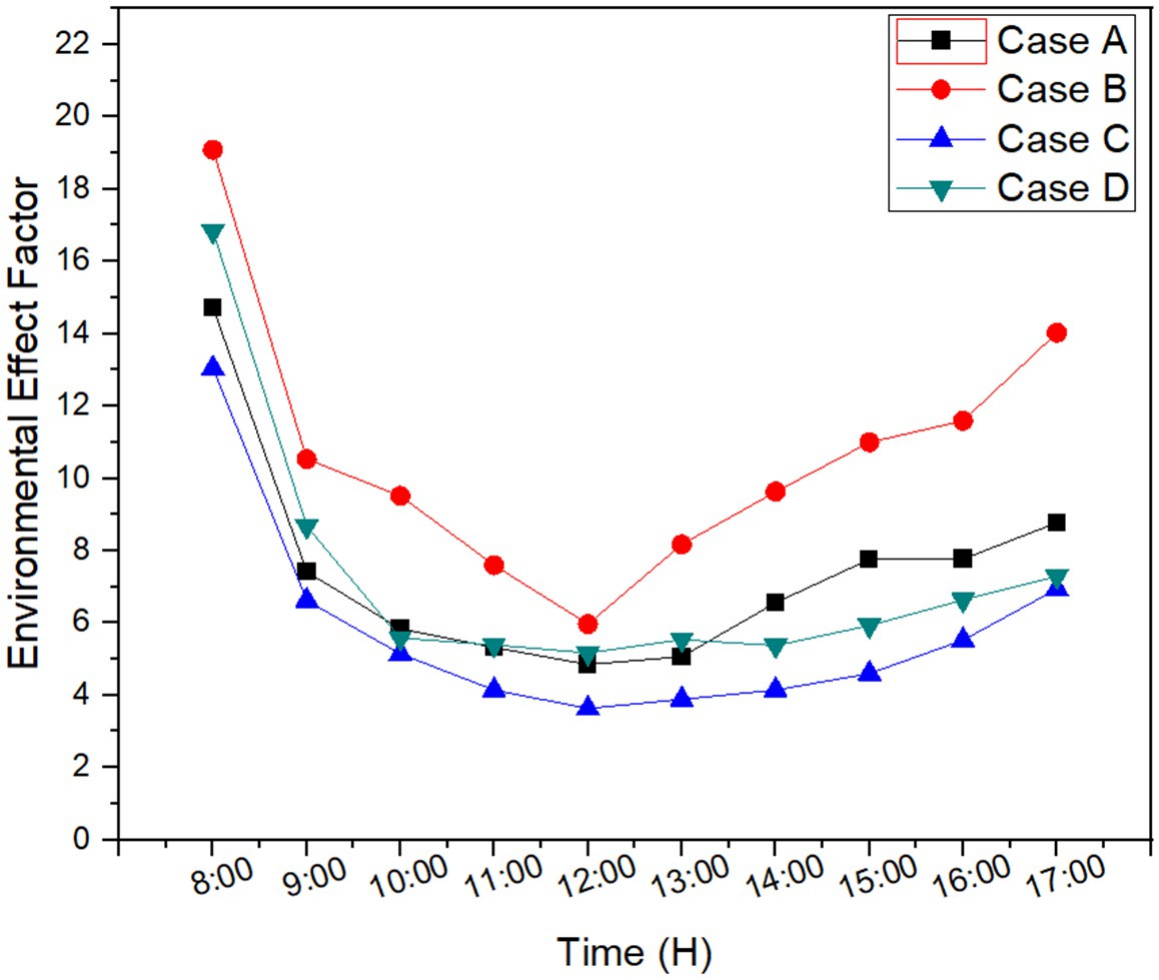
Environmental effect factor for each case.

Environmental effect factor determines the impact of waste exergy on the environment and is calculated using waste energy and exergy efficiency. It can be seen in Fig 7 that case B showed maximum influence on the environment. However, minimum influence was seen for case C. Furthermore, minimum influence for each case was at noon when solar intensity was at peak during the day.

**Fig 8:**
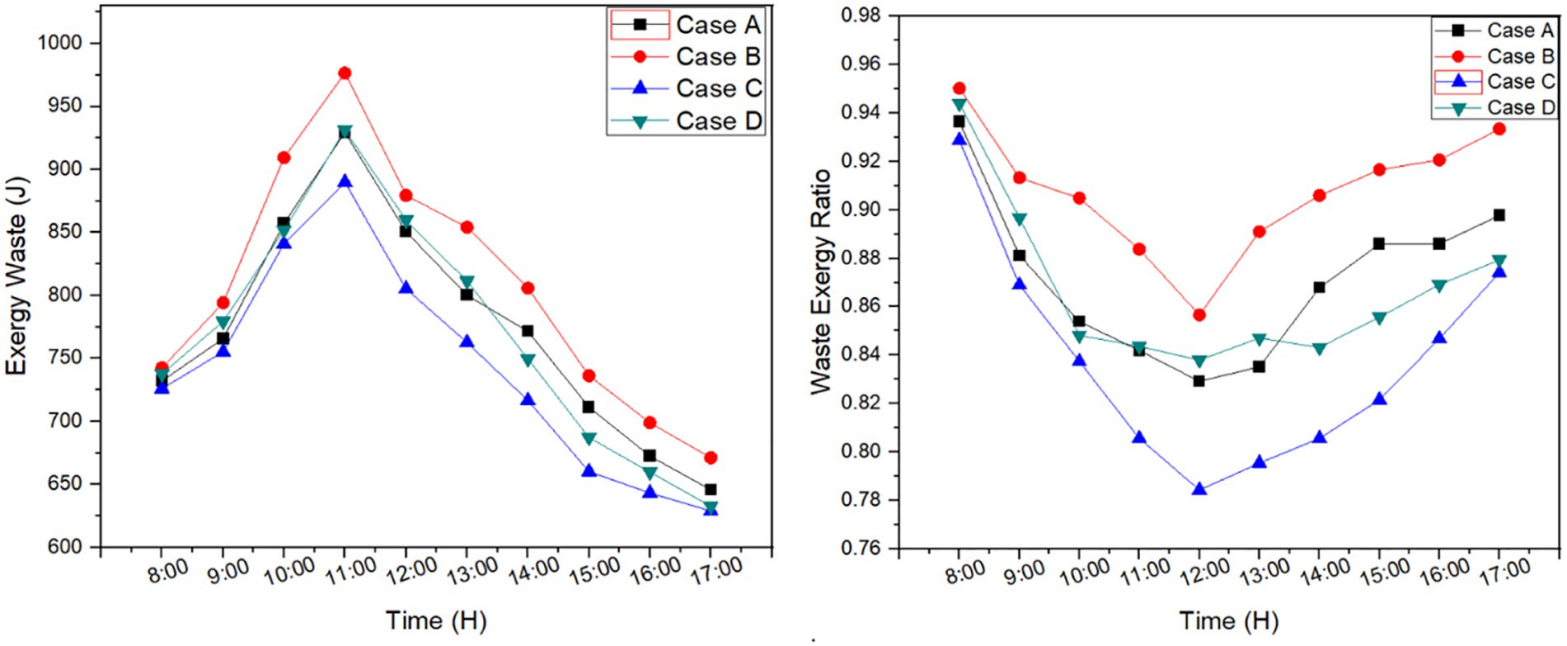
Exergy waste and exergy waste ratio for each case.

Exergy waste is determined as the amount of energy loss or destroyed during the desalination process. A part of it can be recovered and other remained as unrecoverable. Therefore, it is the difference between exergy input and exergy output during the experimentation process. Maximum peak exergy waste was for case B as 976 J and lowest was for case C as 890 J. Furthermore, it was similar for case A and case D which can be seen in Fig 8. However, the addition of external reflector in 2cm basin depth resulted in 4.9% decrease in exergy waste and reduction of basin depth resulted in 5% decrease in exergy waste. The optimal system was defined as case C in exergy waste results which showed 9.7% reduction in exergy waste compared to case B.

Waste exergy ratio is the term used to determine the exergy input which is not found in the system. Fig 8 illustrates waste exergy ratio of each case during the experimentation. It can be seen that maximum waste exergy ratio was for case B and minimum ratio was for case C.

**Fig 9:**
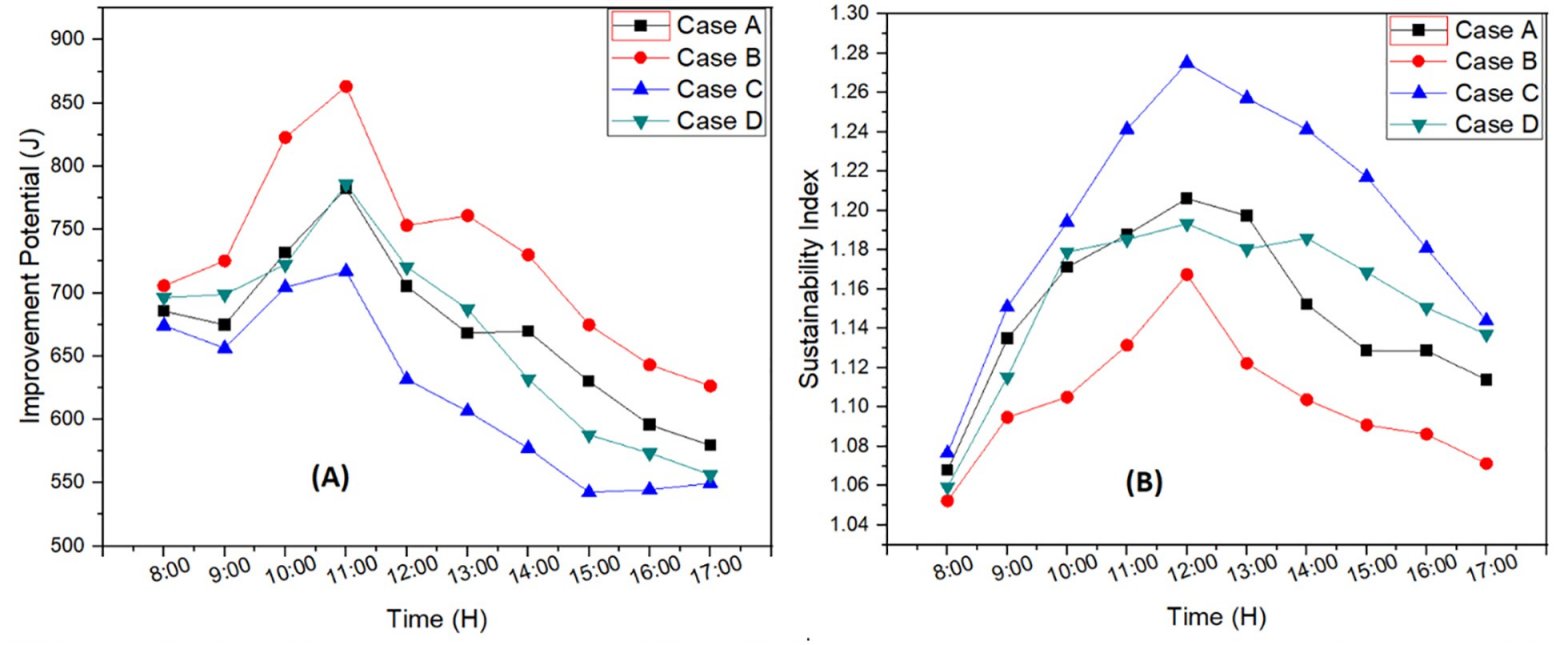
Improvement potential (A) and sustainability index (B) for each case.

Improvement potential term refers to the system enhancement capability or the extent to which system performance can be increased to get full use of available energy. Fig 9 shows the improvement potential for each case. Hence, it can be seen maximum improvement was needed at 11:00 AM which indicated high loss of energy. It was clearly depicted from the Fig 9 that case B i.e., solar still with 4cm basin depth and internal reflector requires more improvement. However, with the addition of external reflector to this case, the output results were enhanced, and it can be seen from the Fig 9 for case D. Furthermore, system was further enhanced by reducing the depth of basin to 2cm and case C represents that system which showed minimum improvement potential required in this study. Maximum peak improvement potential of 863 J was measured for case B and minimum peak improvement potential was 717 J for case C. For case A and case D, maximum improvement potential was almost same as 784 J. The decrease in improvement potential in optimal case was 16.9% by reducing the basin depth to 2cm and adding external reflector in solar still system.

Solar still performance was further analyzed in terms of sustainability index. This term is derived from exergy efficiency and indicates the performance of the system. Fig 9 shows the sustainability index of each case. It can be seen that the optimal system had high sustainability index compared to other cases. Maximum peak sustainability index was calculated as 1.27 for case C followed by case A as 1.20, case D as 1.19 and case B as 1.16. It can be observed that as the solar intensity was increased, system gained high sustainability. Thus, solar still with 2cm basin depth and external reflector performance was almost the similar as solar still with 4cm basin depth and both reflectors. The increase in sustainability index for optimal system was 9.48% which was caused due to addition of external reflector and decrease in basin depth. Sustainability index of case C represented the most sustainable system in the experimentation.

### 3.6 Sensitivity analysis

**Fig 10:**
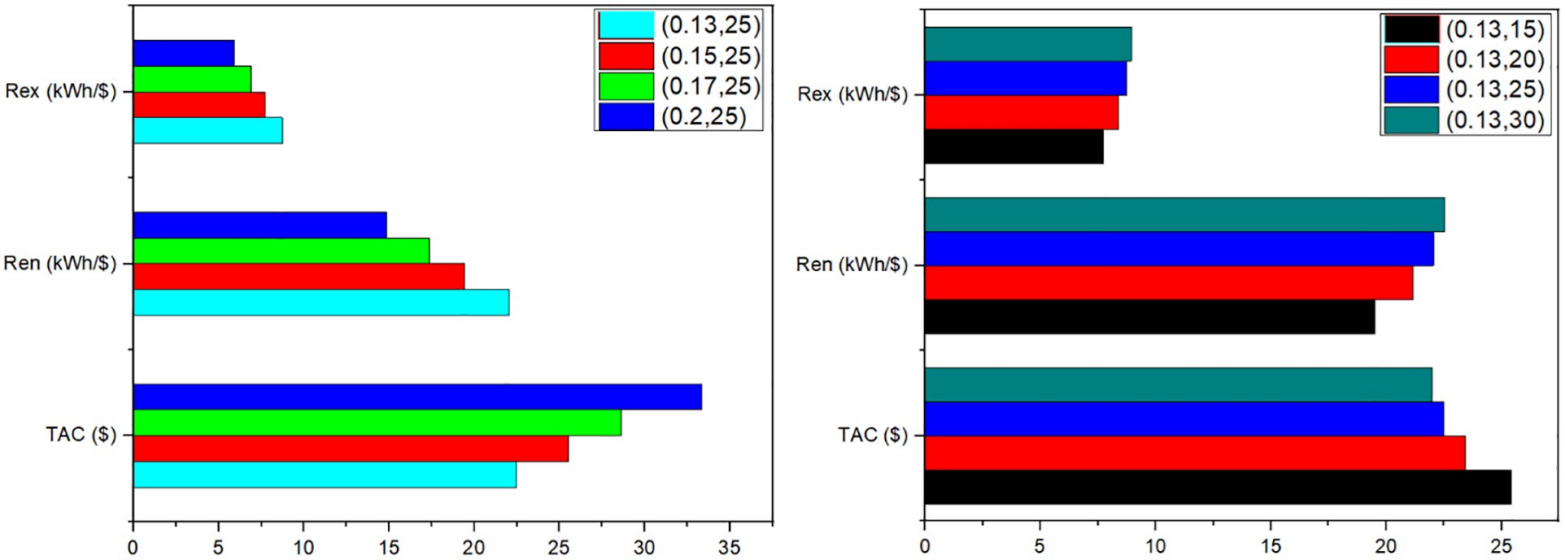
Sensitivity analysis for optimal case, (A) Change in interest rate; (B) Change in lifetime.

Sensitivity analysis is performed to intercept the future predictions and check the feasibility of the system considering the change in parameters which can directly affect the system cost and performance. In this study, two variables were checked for fully analyzing the performance of solar still system. First was the change in interest rate and second was the total lifetime of the system. As interest rate and lifetime can be varied for each future system so the system performance for possible change in interest rate have been analyzed during sensitivity analysis. Fig 10 shows the sensitivity analysis results on an optimal solar still system in this study. In part A, interest rate was varied from 0.13% to 0.2% while the life of solar still was remained constant as 25 years. In part B, lifetime of solar still was varied from 15 years to 30 years while keeping interest rate constant as 0.13%. Lowering interest rate and increasing lifetime results in better output in optimal system. Moreover, it was predicted that change in interest rate greatly effects the exergo-economic parameters compared to change in solar still life time.

### 3.7 Environmental impact analysis

Environmental impact assessment was undertaken to measure the carbon dioxide reduction due to use of solar still for clean water. Net carbon dioxide mitigation for the whole life of optimal solar still was calculated as 23.91 tons. Table 3 shows the results of environmental analysis parameters for optimal system used in this study to evaluate the environmental impact deeply. For enviro-economic analysis, international carbon price was taken as 14.5$ per ton. Thus, the total price for carbon dioxide mitigation during solar still lifetime was 346.7$. However, carbon credits gained in single slope solar still study undertaken by Yousef et al. [33] was 226.6$ and 208.9$ with steel fibers and hollow cylindrical pin fins respectively. The present study carbon dioxide mitigation was also greater when compared to the results of Parsa et al. [36] while considering 20 years of solar still lifetime. Furthermore, exergo-environmental analysis was conducted to measure the comparison of net reduction in carbon emissions using renewable resources and use of fossil fuels for the same productivity. The results showed 8.95 tons of carbon emissions reduction with the use of renewable resource i.e., solar still in this study. It is higher than the passive solar still systems as demonstrated by Parsa et al. [36] however, lower than study conducted by Yousef et al. [33]. The cost reduction considering international carbon price was calculated for solar still life time while performing exergo-enviro-economic analysis which showed 129.8$ cost saving.

**Table 3:** Environmental analysis results for optimal case.

Environmental Analysis	*CO*_*2*_ *emission mitigation*	*Enviro-economic analysis*	*Exergo-environmental analysis*	*Exergo-enviro-economic analysis*
*Environmental parameters*	$\emptyset_{CO_{2}}$	$Z_{CO_{2}}$	$\emptyset_{ex,CO_{2}}$	$Z_{ex,CO_{2}}$
*Optimal systems results*	23.91	346.71	8.95	129.85

### 3.8 Validation

Results achieved from this study were compared with the work of other researchers using solar desalination. It was seen that the results acquired from optimal system in this study were superior. Maximum energy efficiency in our study was 60.19% however it was 56.62% in study performed by Agrawal and Rana [41]. Both studies have been conducted in summer season with single slope solar still. Energy efficiency achieved during experimentation was more when compared Kaviti et al. [49] study of double slope solar still with maximum efficiency of 54.11% using aluminum truncated conic fins. Same goes with the study conducted by Khafaji et al. [47] who achieved maximum energy efficiency of 47.02% with single slope solar still at 1cm depth. Similar increasing trends were achieved by decreasing the depth of solar still as observed in this study. Furthermore, the productivity of studied solar still was 1.34 times more than the single slope solar still undertaken by Hansen et al. [50] with fin based absorber.

## 4. Conclusion

Single slop solar still integrated with both internal and external reflectors and finned plate absorber has experimentally investigated at the climatic conditions of Pakistan. Four cases were studied with case A using only internal reflectors at depth of 2 cm, case B using both internal and external reflectors at depth of 2 cm, case C using only internal reflectors at depth of 4 cm and case D using both internal and external reflectors at depth of 4 cm. Detailed analysis were taken under consideration which involved energy, exergy, enviro-economic, exergy-economic, exergo-enviromental, exergo-enviro-economic, sensitivity and sustainability (6E,2S) analysis. Following results were achieved:

1. The optimum productivity of 7.02 liters has been achieved at depth of 2 cm using both internal and external reflectors i.e., case C (2cm depth with both reflectors).
2. For optimal system, water production cost was 0.033$ per liter while considering 10 years solar still lifetime.
3. Maximum energy and exergy efficiency for optimal system (case C) achieved were 60.19% and 21.57% respectively.
4. Uniform annual cost for optimal system was 22.48 $. The exergo-economic parameter for energy and exergy were determined as 22.05 $ and 8.74 $ respectively for case C.
5. Minimum payback time period for optimal system was 0.88 and 2.23 years for energy and exergy output respectively.
6. Maximum peak sustainability index was calculated as 1.27 for case C which determined best performance of system and maximum improvement potential was 863 J for case B which indicated maximum system enhancement capability.
7. For optimal system, total reduction in carbon dioxide using solar desalination during solar still lifetime was 8.95 tons which resulted in 129.8$ cost saving derived from exergo-environmental and exergo-enviro-economic analysis.
8. For optimal case, annual yield was considered to be 2106 litres while considering 300 bright shiny days.
9. Integrating solar still for areas where there is lack of clean water availability can result in reduction of plastics in the environment.

In this study, sensitivity analysis helped to determine the future predictions and feasibility of the system with the change in parameters i.e., interest rate and total lifetime which resulted in direct influence to the system cost and performance.

### Future directions

It would be interesting to increase the condensation rate by providing condenser at the covering glass and work on phase change material to increase the daily output. In addition to this, the feasibility of the tracking system for the sun must be studied in future work. Furthermore, heater operated by solar photovoltaic panel can be installed in absorber plate to increase the basin water temperature. Such type of solar still by arranging in series or parallel can be effectively utilized for the communal demand of fresh water which will be studied in future.

## Abbreviations

**Nomenclature**

*Parameters*

A: surface area (m^2^)
G: solar irradiation (W m^-2^)
k: thermal conductivity
m: mass (g)
T: temperature (K)
SI: sustainability index
IP: improvement potential
EPF: Energy production factor
EPBT: Energy payback time
TAC: Total annual cost

*Subscripts*

amb: Ambient
w: water
Ex: exergy
En: energy

*Greeks*

${\dot{m}}_{w}$: fresh water production
ƞ_*ex*_: efficiency
$\dot{\text{E}}n$: energy
$\dot{\text{E}}x$: exergy
*R*_*Ex*_: exergy exergo-economic
*R*_*En*_: energy exergo-economic
$Z_{CO_{2}}$: enviro-economic parameter
$X_{CO_{2}}$: international carbon price
$\emptyset_{ex,CO_{2}}$: exergo-environmental parameter
$Z_{ex,CO_{2}}$: exergo-enviro-economic parameter
